# Supplementary material for: The DNA methylation landscape of five pediatric-tumor types
Source: PeerJ. 2022 Jun 10;10:e13516. doi: 10.7717/peerj.13516 (PMC9190670; doi:10.7717/peerj.13516)
Supplement: Supplemental Information 1 [file peerj-10-13516-s001.docx]

# The DNA methylation landscape of five pediatric-tumor types

Alyssa C. Parker^1^, Badi I. Quinteros^1^, Stephen R. Piccolo^1,*^

1 - Department of Biology, Brigham Young University, Provo, UT, USA

* - Please address correspondence to S.R.P. at stephen_piccolo@byu.edu.

# Supplementary Tables

**Table S1: Frequency of methylation level/variation categories for genes in normal pediatric tissues.** For each normal dataset, we determined whether each gene had low, medium, or high methylation levels and whether the coefficient of variation was low or high. This table shows the frequency with which we observed each combination of these categories.

| **Dataset** | **Low methylation / low variation** | **Low methylation / high variation** | **Medium methylation / low variation** | **Medium methylation / high variation** | **High methylation / low variation** | **High methylation / high variation** |
| --- | --- | --- | --- | --- | --- | --- |
| GSE109446 | 12582 (65.59%) | 268 (1.40%) | 3023 (15.76%) | 8 (0.04%) | 3303 (17.22%) | 0 (0.0%) |
| GSE65163 | 12593 (65.64%) | 76 (0.40%) | 3317 (17.29%) | 6 (0.03%) | 3192 (16.64%) | 0 (0.0%) |
| GSE69502 | 11298 (58.89%) | 1696 (8.84%) | 2714 (14.15%) | 143 (0.75%) | 3325 (17.33%) | 8 (0.04%) |
| GSE89278 | 12835 (66.90%) | 82 (0.43%) | 2434 (12.69%) | 4 (0.02%) | 3829 (19.96%) | 0 (0.0%) |

**Table S2: Probes for which methylation levels differed between tumors and normal tissues.** We compared methylation levels at the probe level between each pediatric tumor type and the normal datasets. This table lists probes that resulted in a Bejamini-Hochberg False Discovery Rate of 0.05 or smaller and an absolute, log2-transformed fold change of 2 or greater.

| **Probe** | **Gene** | **CCSK** | **NBL** | **OS** | **RT** | **WT** |
| --- | --- | --- | --- | --- | --- | --- |
| cg00092518 | PHF11 | 2.69 | 0.83 | 0.54 | -0.19 | 1.04 |
| cg10982364 | SATB2 | 2.54 | -0.32 | 1.05 | -0.32 | 0.37 |
| cg02685896 | PROCA1 | 2.47 | 0.57 | 0.05 | 0.21 | 0.58 |
| cg27183188 | ALDOC | 2.47 | 0.56 | 1.44 | 1.61 | 0.90 |
| cg16484943 | PLA2G16 | 2.45 | 0.18 | 0.08 | 0.07 | 0.19 |
| cg13044675 | LTBP3 | 2.45 | 0.16 | 2.14 | 0.77 | 0.07 |
| cg04360793 | ELTD1 | 2.43 | 1.33 | 0.73 | 1.33 | 1.43 |
| cg13906823 | TSTD1 | 2.38 | 1.26 | 2.63 | 2.25 | 0.28 |
| cg15202102 | PRKCDBP | 2.38 | 1.26 | -0.09 | 0.87 | 1.82 |
| cg27180868 | TSTD1 | 2.35 | 1.11 | 2.28 | 1.89 | 0.32 |
| cg01074657 | GIPC2 | 2.34 | 0.21 | 1.53 | 1.17 | 1.73 |
| cg24161057 | TSTD1 | 2.34 | 1.17 | 2.54 | 2.04 | 0.22 |
| cg16565913 | RBM47 | 2.34 | 0.90 | 2.13 | 1.26 | 0.17 |
| cg05638689 | LTBP3 | 2.34 | 0.24 | 1.89 | 1.24 | 0.21 |
| cg15219506 | PLA2G16 | 2.33 | 0.00 | 0.06 | 0.13 | 0.10 |
| cg13356117 | TTC12 | 2.29 | 0.07 | 0.38 | 0.02 | 0.07 |
| cg19697239 | NINJ2 | 2.29 | 0.68 | 0.12 | 1.78 | 0.96 |
| cg14652131 | SPON2 | 2.28 | 0.07 | 0.96 | 1.66 | 0.23 |
| cg26678920 | PRKCDBP | 2.28 | 1.30 | -0.06 | 0.92 | 1.80 |
| cg00318756 | IL12RB2 | 2.24 | 0.10 | 0.91 | 1.12 | 0.06 |
| cg09721739 | NMNAT3 | 2.22 | 1.55 | 0.28 | 0.90 | 1.56 |
| cg23363832 | RBP1 | 2.22 | -0.02 | 0.30 | 1.30 | -0.13 |
| cg22539420 | SATB2 | 2.21 | -0.03 | 1.00 | 0.11 | 0.36 |
| cg06235847 | PLA2G16 | 2.21 | 0.22 | 0.08 | -0.05 | 0.12 |
| cg09282497 | KCNS3 | 2.20 | 0.15 | 0.37 | -0.24 | 0.18 |
| cg12426196 | TSTD1 | 2.19 | 1.32 | 2.30 | 2.05 | 0.30 |
| cg19725903 | PLBD1 | 2.18 | 0.61 | 0.06 | 0.05 | 0.67 |
| cg01240734 | AIFM2 | 2.18 | 0.13 | 0.05 | 0.71 | -0.05 |
| cg15084543 | ELTD1 | 2.16 | 1.25 | 0.65 | 1.01 | 1.27 |
| cg07281879 | WT1 | 2.12 | 0.18 | 0.29 | 1.17 | 0.52 |
| cg23693289 | PTK2B | 2.10 | 1.88 | 0.07 | 0.61 | 0.06 |
| cg24841318 | PROCA1 | 2.10 | 0.63 | -0.08 | -0.10 | 0.64 |
| cg05165940 | SATB2 | 2.10 | -0.45 | 0.89 | 0.08 | 0.43 |
| cg06671340 | DENND1B | 2.09 | 0.51 | 1.06 | 0.96 | 0.43 |
| cg08032924 | CMTM2 | 2.08 | 0.16 | 1.92 | 1.86 | 0.09 |
| cg08786077 | QPCT | 2.08 | 0.27 | 0.79 | 0.26 | 1.56 |
| cg14621053 | ADAM12 | 2.08 | 0.28 | 0.39 | 0.46 | 0.16 |
| cg09234616 | WT1 | 2.07 | -0.35 | 0.30 | 1.43 | 0.12 |
| cg14668747 | ALDOC | 2.05 | 0.24 | 1.17 | 0.54 | 0.54 |
| cg05237641 | ADAM12 | 2.04 | 0.13 | 0.50 | 0.21 | 0.17 |
| cg08332594 | DMPK | 2.04 | 0.92 | 1.58 | 0.30 | 1.23 |
| cg25486143 | RASSF1 | 2.03 | 1.99 | -0.14 | 1.82 | 2.20 |
| cg26422458 | ELTD1 | 2.03 | 1.03 | 0.52 | 1.05 | 1.16 |
| cg01832036 | DLX4 | 2.03 | -0.59 | 1.61 | 1.06 | 0.42 |
| cg27068490 | TTC12 | 2.02 | 0.03 | 0.17 | -0.14 | 0.07 |
| cg15162392 | QPCT | 2.02 | 0.00 | 0.65 | -0.22 | 1.40 |
| cg24157171 | TCF21 | 2.02 | -0.26 | 0.01 | 0.29 | 0.19 |
| cg24984523 | PHF11 | 2.01 | 0.74 | 0.19 | 0.23 | 0.83 |
| cg00049664 | CMTM2 | 2.01 | -0.01 | 1.54 | 1.32 | -0.31 |
| cg13466383 | TSTD1 | 1.96 | 1.02 | 2.07 | 1.42 | 0.32 |
| cg16179125 | CTSZ | 1.85 | 2.26 | 0.60 | 2.24 | 1.34 |
| cg23445461 | PCDHGC4 | 1.85 | 0.24 | 2.23 | 2.07 | 0.43 |
| cg07834574 | C17orf107 | 1.81 | 0.80 | 1.12 | 2.01 | 2.35 |
| cg09527362 | MB21D1 | 1.73 | 1.47 | -0.11 | 0.54 | 2.01 |
| cg13050240 | RIPK3 | 1.71 | 1.09 | 1.16 | 1.81 | 2.01 |
| cg24713667 | MB21D1 | 1.70 | 1.35 | -0.24 | 0.55 | 2.19 |
| cg05525106 | MB21D1 | 1.67 | 1.51 | -0.17 | 0.53 | 2.16 |
| cg04420309 | RHOD | 1.66 | 0.79 | 0.76 | 2.04 | 0.08 |
| cg14819504 | LY75 | 1.66 | 0.72 | 0.61 | 1.59 | 2.48 |
| cg20814095 | C17orf107 | 1.54 | 0.65 | 0.96 | 2.03 | 2.26 |
| cg07482202 | FBXL16 | 1.53 | 2.04 | 0.07 | 0.17 | -0.18 |
| cg14275842 | C17orf107 | 1.51 | 0.55 | 0.81 | 1.90 | 2.20 |
| cg15120454 | RHBG | 1.48 | 1.43 | -0.04 | 2.07 | 0.91 |
| cg14482741 | C17orf107 | 1.44 | 0.60 | 0.99 | 1.84 | 2.05 |
| cg01092293 | LY75 | 1.44 | 1.05 | 0.85 | 1.57 | 2.20 |
| cg20758982 | MB21D1 | 1.28 | 1.11 | -0.30 | 0.38 | 2.00 |
| cg15693066 | RHOD | 1.27 | 0.95 | 0.74 | 2.06 | 0.08 |
| cg09902229 | CTSZ | 1.27 | 2.07 | 0.37 | 2.03 | 1.00 |
| cg01311360 | PCDHGA10 | 1.21 | 1.15 | 1.06 | 1.15 | 2.11 |
| cg26904406 | TRAF3IP2 | 1.19 | 2.13 | 0.00 | 1.10 | 0.08 |
| cg10917547 | PCDHGA10 | 1.17 | 1.27 | 1.08 | 1.38 | 2.13 |
| cg08828819 | PON1 | 1.15 | 0.79 | 0.32 | 1.72 | 2.66 |
| cg06499647 | C2orf40 | 1.14 | -0.06 | 0.10 | 0.57 | 2.07 |
| cg02280532 | LY75 | 1.12 | 0.34 | 0.20 | 1.66 | 2.36 |
| cg22859061 | SCGB3A1 | 1.12 | -0.25 | -0.01 | 2.23 | 2.00 |
| cg10147797 | SDR42E1 | 1.11 | 2.02 | 0.24 | 1.11 | 1.29 |
| cg23049458 | L1TD1 | 1.09 | 1.64 | 0.89 | 0.98 | 2.53 |
| cg07121856 | PON1 | 0.98 | 0.83 | 0.41 | 1.47 | 2.27 |
| cg18514922 | LY75 | 0.97 | 0.48 | 0.27 | 1.57 | 2.27 |
| cg00237391 | DEF6 | 0.90 | 2.20 | 2.03 | 1.32 | -0.09 |
| cg21627409 | PCDHGA12 | 0.88 | 1.04 | 0.99 | 1.80 | 2.53 |
| cg06418871 | MARVELD2 | 0.84 | 2.01 | 1.64 | 1.13 | -0.19 |
| cg26010734 | EPHX3 | 0.80 | 0.79 | 1.72 | 2.05 | 2.10 |
| cg02452944 | PCDHGA12 | 0.65 | 0.76 | 0.81 | 1.60 | 2.34 |
| cg18556834 | REC8 | 0.59 | 0.18 | 2.02 | 1.76 | 0.30 |
| cg22847228 | PCDHGA10 | 0.57 | 0.84 | 0.96 | 1.28 | 2.04 |
| cg11260046 | MEI1 | 0.52 | 0.45 | 0.46 | 1.06 | 2.19 |
| cg19989295 | REC8 | 0.49 | -0.25 | 2.16 | 1.96 | 0.08 |
| cg17399362 | EPHX3 | 0.47 | 0.50 | 1.50 | 1.75 | 2.04 |
| cg16425038 | REC8 | 0.43 | -0.14 | 2.23 | 1.79 | 0.02 |
| cg22861116 | PCDHGA7 | 0.42 | 0.58 | 1.16 | 1.60 | 2.34 |
| cg06747432 | CCDC8 | 0.29 | 2.01 | 0.43 | 0.11 | -0.02 |
| cg16847637 | CPA1 | 0.15 | -1.11 | -0.55 | -1.23 | -2.16 |
| cg05020685 | APBB2 | 0.13 | 0.58 | 2.21 | 0.76 | 0.23 |
| cg12455762 | APBB2 | 0.06 | 0.34 | 2.02 | 0.19 | 0.23 |
| cg08668790 | ZNF154 | 0.02 | 0.55 | 1.26 | -0.14 | 2.01 |
| cg12506930 | ZNF154 | -0.02 | 0.41 | 1.23 | -0.12 | 2.04 |
| cg07654934 | LXN | -0.06 | 2.23 | 1.56 | 1.05 | 0.43 |
| cg06485940 | APBB2 | -0.07 | -0.14 | 2.10 | 0.64 | -0.01 |
| cg27049766 | ZNF154 | -0.07 | 0.57 | 1.26 | -0.11 | 2.04 |
| cg10817615 | CHST1 | -0.08 | 0.06 | -0.04 | 2.02 | 0.07 |
| cg13912673 | C9orf64 | -0.10 | -0.15 | -0.09 | 0.46 | 2.36 |
| cg04917391 | APBB2 | -0.12 | -0.01 | 2.00 | -0.22 | 0.14 |
| cg14140881 | EFCAB10 | -0.14 | -0.09 | 0.94 | 2.08 | -0.03 |
| cg18781988 | PCDHGA3 | -0.15 | 0.01 | 0.53 | 1.21 | 2.11 |
| cg23735712 | APBB2 | -0.15 | 0.04 | 2.55 | 0.55 | 0.03 |
| cg21211480 | TMEM106A | -0.16 | 1.12 | -0.05 | 2.03 | 0.19 |
| cg21790626 | ZNF154 | -0.18 | 0.10 | 1.09 | -0.48 | 2.12 |
| cg18222083 | TMEM106A | -0.20 | 1.41 | -0.11 | 2.34 | 0.25 |
| cg15140703 | STAG3 | -0.20 | -0.21 | 0.60 | 0.07 | 2.07 |
| cg05921905 | HOXA2 | -0.21 | -0.31 | 2.01 | 0.26 | 0.29 |
| cg19018201 | FGF6 | -0.30 | -1.37 | -0.17 | -0.29 | -2.24 |
| cg24965248 | FGF6 | -0.30 | -1.49 | -0.35 | -0.55 | -2.47 |
| cg10253847 | NKAPL | -0.64 | -0.54 | 0.18 | 1.45 | 2.08 |
| cg15013801 | ASCC1 | -1.05 | -1.58 | -0.62 | -1.76 | -2.53 |
| cg17372758 | ASCC1 | -1.18 | -1.59 | -0.73 | -2.12 | -2.29 |
| cg25135457 | COASY | -1.35 | -0.80 | -1.11 | -1.04 | -2.06 |
| cg24881255 | RAX2 | -1.94 | -1.51 | -0.87 | -0.77 | -2.14 |
| cg14871736 | NMNAT3 | -2.02 | -0.36 | -1.20 | -1.18 | -1.16 |
| cg13508402 | LRRC4 | -2.02 | -0.91 | 0.50 | -0.52 | 0.11 |
| cg24767968 | HOXB3 | -2.03 | -1.28 | -0.35 | -0.93 | -1.18 |
| cg24964110 | CABLES1 | -2.04 | -0.73 | -0.73 | -0.39 | -1.34 |
| cg08124030 | TM4SF1 | -2.05 | 0.01 | -0.95 | -0.46 | 0.22 |
| cg03779374 | ZBTB20 | -2.10 | -0.64 | -1.85 | -0.04 | -1.28 |
| cg22369786 | SPRY2 | -2.15 | 0.00 | 0.57 | -0.46 | -1.52 |
| cg12910797 | HOXB3 | -2.17 | -1.40 | -0.25 | -1.14 | -1.56 |
| cg20566840 | HCAR1 | -2.21 | -0.37 | -1.26 | -0.68 | -2.01 |
| cg22972858 | HCAR1 | -2.21 | -0.46 | -1.28 | -0.78 | -2.10 |
| cg21266845 | DPT | -2.22 | -0.20 | -1.55 | -0.44 | -0.14 |
| cg09922481 | DPT | -2.23 | -0.47 | -1.49 | -0.94 | -0.32 |
| cg24422029 | CACNB2 | -2.23 | -0.62 | -0.74 | -0.76 | -1.39 |
| cg06714705 | OLFML1 | -2.25 | -0.43 | -0.75 | -1.28 | -1.55 |
| cg13313047 | CABLES1 | -2.28 | -0.78 | -0.79 | 0.02 | -1.28 |
| cg26136497 | CABLES1 | -2.28 | -1.00 | -0.64 | -0.32 | -1.45 |
| cg13293524 | HOXB3 | -2.36 | -1.65 | -0.43 | -1.50 | -1.61 |
| cg04117801 | HOXB3 | -2.36 | -1.32 | -0.47 | -0.97 | -1.78 |
| cg13280788 | HOXB3 | -2.38 | -1.54 | -0.76 | -1.25 | -1.54 |
| cg19344626 | NWD1 | -2.40 | -1.28 | -1.51 | -0.59 | -0.52 |
| cg13702536 | HCAR1 | -2.49 | -0.31 | -1.40 | -0.55 | -2.30 |

**Table S3: Genes for which methylation levels differed between tumors and normal tissues.** We compared methylation levels at the gene level between each pediatric tumor type and the normal datasets. This table lists genes that resulted in a Bejamini-Hochberg False Discovery Rate of 0.05 or smaller and an absolute, log2-transformed fold change of 2 or greater.

| **Entity** | **CCSK** | **NBL** | **OS** | **RT** | **WT** |
| --- | --- | --- | --- | --- | --- |
| PLA2G16 | 2.53 | 0.28 | 0.07 | 0.13 | 0.20 |
| TSTD1 | 2.52 | 1.35 | 2.69 | 2.18 | 0.38 |
| SATB2 | 2.32 | -0.21 | 1.03 | -0.04 | 0.35 |
| PROCA1 | 2.14 | 0.79 | 0.00 | -0.16 | 1.12 |
| PRKCDBP | 2.09 | 1.11 | -0.23 | 0.68 | 1.64 |
| CMTM2 | 2.05 | 0.23 | 1.83 | 1.76 | 0.03 |
| DENND1B | 2.03 | 0.45 | 0.85 | 0.80 | 0.34 |
| DRD4 | 2.02 | -0.10 | 0.71 | 1.82 | 0.01 |
| QPCT | 2.02 | 0.31 | 0.72 | 0.09 | 1.55 |
| MB21D1 | 1.70 | 1.43 | -0.20 | 0.40 | 2.27 |
| C17orf107 | 1.61 | 0.69 | 1.02 | 2.03 | 2.35 |
| PCDHGC4 | 1.58 | 0.18 | 2.21 | 2.06 | 0.41 |
| C2orf40 | 1.37 | 0.08 | 0.18 | 0.58 | 2.06 |
| LY75 | 1.25 | 0.76 | 0.60 | 1.62 | 2.45 |
| PCDHGA10 | 0.88 | 1.10 | 1.08 | 1.35 | 2.15 |
| LRRC8E | 0.82 | 0.03 | 0.03 | 2.07 | 0.28 |
| PCDHGA12 | 0.59 | 0.94 | 0.86 | 1.60 | 2.22 |
| REC8 | 0.42 | -0.02 | 2.11 | 1.81 | 0.09 |
| ARHGDIB | 0.32 | 0.24 | 0.70 | 1.18 | 2.12 |
| HOXD3 | 0.27 | 2.22 | 2.11 | 1.52 | 1.37 |
| PCDHGA7 | 0.18 | 0.55 | 0.94 | 1.47 | 2.09 |
| CCDC8 | 0.11 | 2.13 | 0.40 | -0.01 | -0.41 |
| APBB2 | -0.01 | 0.19 | 2.43 | 0.45 | 0.14 |
| ZNF154 | -0.05 | 0.43 | 1.39 | -0.17 | 2.26 |
| TMEM106A | -0.13 | 1.34 | -0.06 | 2.09 | 0.33 |
| SUPT3H | -0.14 | 0.04 | 2.02 | -0.35 | -0.02 |
| EML2 | -0.21 | 2.01 | -0.09 | -0.12 | -0.03 |
| ASCC1 | -1.07 | -1.55 | -0.63 | -2.00 | -2.51 |
| HCAR1 | -2.05 | -0.35 | -1.18 | -0.59 | -1.90 |
| PXDNL | -2.12 | 0.00 | -1.31 | -0.12 | -1.90 |
| SPRY2 | -2.21 | 0.04 | 0.68 | -0.46 | -1.55 |
| DPT | -2.31 | -0.37 | -1.60 | -1.01 | -0.21 |

**Table S4: Genes for which methylation levels differed significantly among pediatric tumor types.** We contrasted methylation levels between each pair of tumor types and identified genes for which mean methylation values differed significantly between at least two tumor types. This table shows genes that resulted in a False Discovery Rate (FDR) of 0.05 or smaller and had an absolute, log2-transformed fold change of 2 or greater. CCSK = clear cell sarcomoa of the kidneys; NBL = neuroblastomas; OS = osteosarcomas; RT = rhabdoid tumors; WT = Wilms tumors.

| **Gene** | **CCSK vs. NBL** | **CCSK vs. OS** | **CCSK vs. RT** | **CCSK vs. WT** | **NBL vs. OS** | **NBL vs. RT** | **NBL vs. WT** | **OS vs. RT** | **OS vs. WT** | **RT vs. WT** |
| --- | --- | --- | --- | --- | --- | --- | --- | --- | --- | --- |
| SPRY2 | -2.22 | -2.86 | -1.72 | -0.62 | -0.65 | 0.50 | 1.60 | 1.15 | 2.25 | 1.10 |
| NKAPL | -0.47 | -1.09 | -2.11 | -2.70 | -0.62 | -1.64 | -2.23 | -1.02 | -1.61 | -0.59 |
| CKMT2 | 0.97 | 0.83 | 0.48 | 2.55 | -0.14 | -0.49 | 1.57 | -0.35 | 1.71 | 2.06 |
| CCDC8 | -2.07 | -0.33 | 0.06 | 0.46 | 1.73 | 2.13 | 2.53 | 0.40 | 0.79 | 0.40 |
| SATB2 | 2.48 | 1.23 | 2.30 | 1.90 | -1.24 | -0.18 | -0.58 | 1.07 | 0.67 | -0.40 |
| MB21D1 | 0.27 | 1.91 | 1.31 | -0.57 | 1.64 | 1.04 | -0.83 | -0.60 | -2.48 | -1.88 |
| HOXA5 | -0.69 | -2.48 | -1.44 | -0.59 | -1.78 | -0.74 | 0.10 | 1.04 | 1.88 | 0.85 |
| PLA2G16 | 2.22 | 2.44 | 2.37 | 2.30 | 0.21 | 0.15 | 0.08 | -0.06 | -0.14 | -0.07 |
| ZNF154 | -0.55 | -1.51 | 0.04 | -2.39 | -0.96 | 0.59 | -1.84 | 1.55 | -0.89 | -2.44 |
| APBB2 | -0.20 | -2.43 | -0.46 | -0.15 | -2.23 | -0.26 | 0.05 | 1.98 | 2.28 | 0.31 |
| TRIM2 | -2.42 | -1.30 | -2.16 | -0.24 | 1.12 | 0.26 | 2.19 | -0.86 | 1.07 | 1.92 |
| SUPT3H | -0.22 | -2.22 | 0.15 | -0.19 | -2.00 | 0.38 | 0.04 | 2.37 | 2.03 | -0.34 |
| HOXA2 | -0.25 | -2.37 | -0.80 | -0.59 | -2.12 | -0.55 | -0.33 | 1.57 | 1.78 | 0.22 |
| PRKCDBP | 0.97 | 2.31 | 1.41 | 0.44 | 1.35 | 0.44 | -0.52 | -0.90 | -1.87 | -0.97 |
| PROCA1 | 1.36 | 2.14 | 2.31 | 1.03 | 0.78 | 0.94 | -0.33 | 0.16 | -1.11 | -1.28 |
| TSTD1 | 1.08 | -0.26 | 0.24 | 2.02 | -1.33 | -0.84 | 0.95 | 0.50 | 2.28 | 1.78 |
| TMEM106A | -1.48 | -0.09 | -2.25 | -0.49 | 1.39 | -0.76 | 1.00 | -2.15 | -0.39 | 1.76 |
| EML2 | -2.22 | -0.12 | -0.09 | -0.18 | 2.09 | 2.13 | 2.04 | 0.04 | -0.06 | -0.09 |
| SULT1C4 | -0.79 | -1.63 | -2.15 | -0.11 | -0.84 | -1.36 | 0.68 | -0.52 | 1.52 | 2.04 |
| REC8 | 0.42 | -1.71 | -1.42 | 0.31 | -2.12 | -1.83 | -0.11 | 0.29 | 2.01 | 1.72 |
| PXDNL | -2.12 | -0.80 | -2.00 | -0.21 | 1.32 | 0.12 | 1.91 | -1.20 | 0.59 | 1.79 |
| DPT | -1.93 | -0.70 | -1.29 | -2.09 | 1.23 | 0.64 | -0.15 | -0.59 | -1.39 | -0.79 |
| DRD4 | 2.08 | 1.28 | 0.16 | 1.97 | -0.80 | -1.92 | -0.12 | -1.12 | 0.69 | 1.80 |
| NAV1 | -0.07 | -1.63 | -0.02 | 0.44 | -1.56 | 0.05 | 0.51 | 1.61 | 2.07 | 0.46 |
| ZC3H12D | -0.04 | 1.19 | -0.37 | -0.86 | 1.23 | -0.33 | -0.82 | -1.57 | -2.05 | -0.49 |
| AIFM2 | 1.64 | 2.05 | 1.46 | 2.03 | 0.41 | -0.18 | 0.39 | -0.59 | -0.02 | 0.57 |
| HOXB3 | -0.84 | -2.05 | -1.32 | -0.62 | -1.21 | -0.48 | 0.21 | 0.73 | 1.42 | 0.69 |
| LRRC8E | 0.75 | 0.75 | -1.29 | 0.49 | 0.00 | -2.04 | -0.26 | -2.04 | -0.26 | 1.78 |
| PCDHGC4 | 1.38 | -0.65 | -0.50 | 1.14 | -2.03 | -1.88 | -0.24 | 0.15 | 1.79 | 1.65 |
| SCGB3A1 | 1.22 | 1.01 | -0.81 | -0.74 | -0.21 | -2.02 | -1.96 | -1.82 | -1.75 | 0.07 |
| C10orf11 | -0.30 | -0.26 | 0.81 | 1.70 | 0.04 | 1.11 | 2.00 | 1.07 | 1.97 | 0.90 |

# Supplementary Figures

**Figure S1: Median beta values per probe in normal datasets** We calculated the median beta value per probe across all patients in a given normal dataset. These values are displayed as a histogram.

**Figure S2: Relationship between medium methylation (beta) value and coefficient of variation for each probe.**

**Figure S3: Relationship between medium methylation (beta) value and coefficient of variation for each gene.**

**Figure S4: Methylation levels for the TSTD1 gene under normal conditions and in tumors.** Methylation of TSTD1 was the only gene that showed significantly higher methylation levels than normal cells for three tumor types. CCSK = clear cell sarcoma of the kidneys, NBL = neuroblastomas, OS = osteosarcomas, RT = rhabdoid tumors, WT = Wilms tumors.

**Figure S5: Methylation levels for oncogenes compared to tumor suppressor genes.** We compared mean methylation levels under normal conditions for oncogenes and against levels for tumor suppressor genes. The difference between these two gene categories was not statistically significant.

**Figure S6: Principal component analysis before batch correction.** We used a principal component analysis to look for systematic differences in methylation levels across the datasets. As shown in this plot for the first two principal components, samples from each dataset and/or tissue type typically clustered together.

**Figure S7: Principal component analysis after batch correction.** A principal component analysis revealed that there were systematic differences in methylation levels across the datasets. To support cross-tissue comparisons, we used a linear model to adjust for these differences. This plot shows the first two principal components after performing this correction.
